# Measurement properties of the Minimal Insomnia Symptom Scale (MISS) in an elderly population in Sweden

**DOI:** 10.1186/1471-2318-10-84

**Published:** 2010-11-05

**Authors:** Amanda Hellström, Peter Hagell, Cecilia Fagerström, Ania Willman

**Affiliations:** 1 School of Health Science, Blekinge Institute of Technology, SE-371 79 Karlskrona, Sweden; 2 Department of Health Sciences, Lund University, Lund, Sweden

## Abstract

**Background:**

Insomnia is common among elderly people and associated with poor health. The Minimal Insomnia Symptom Scale (MISS) is a three item screening instrument that has been found to be psychometrically sound and capable of identifying insomnia in the general population (20-64 years). However, its measurement properties have not been studied in an elderly population. Our aim was to test the measurement properties of the MISS among people aged 65 + in Sweden, by replicating the original study in an elderly sample.

**Methods:**

Data from a cross-sectional survey of 548 elderly individuals were analysed in terms of assumptions of summation of items, floor/ceiling effects, reliability and optimal cut-off score by means of ROC-curve analysis and compared with self-reported insomnia criteria.

**Results:**

Corrected item-total correlations ranged between 0.64-0.70, floor/ceiling effects were 6.6/0.6% and reliability was 0.81. ROC analysis identified the optimal cut-off score as ≥7 (sensitivity, 0.93; specificity, 0.84; positive/negative predictive values, 0.256/0.995). Using this cut-off score, the prevalence of insomnia in the study sample was 21.7% and most frequent among women and the oldest old.

**Conclusions:**

Data support the measurement properties of the MISS as a possible insomnia screening instrument for elderly persons. This study make evident that the MISS is useful for identifying elderly people with insomnia-like sleep problems. Further studies are needed to assess its usefulness in identifying clinically defined insomnia.

## Background

According to the International Classification of Sleep Disorders, second edition (ISCD-2), the diagnosis of insomnia is based on a subjective report of difficulties initiating sleep, maintaining sleep due to night time or early morning waking or non-restorative sleep, despite ideal sleep conditions, leading to daytime impairments [1-3]. Secondary insomnia is the most common form and can be related to an underlying somatic or psychic disease or pharmaceutical use [4].

Many elderly people suffer from one or more chronic medical conditions that can cause or contribute to sleep difficulties [5]. Persistent insomnia in elderly persons is often associated with co-morbidity, use of medication or other primary sleep disorders [6]. Common sleep difficulties among elderly people include waking at night and longer periods of wakefulness before falling asleep again [7-9].

Sleep difficulties, such as persistent insomnia, are associated with immune system changes [10], the development of diseases such as depression as well as impaired memory function and ability to concentrate and make decisions [11,12]. Consequently insomnia also has societal and economic implications and is for example associated with an elevated risk of disability pension [13]. However, while a significant minority of the adult population has at least one insomnia symptom occurring three nights or more per week, the majority do not seek help. This can be explained by lack of knowledge about insomnia in the general population but also by under-recognition and under-diagnosis on the part of health care personnel. It is important to take symptoms of insomnia seriously [14], since they tend to become persistent [1].

In general, insomnia has been found to increase with age and is more common among women [7]. In persons aged 65 years or over, the risk of developing insomnia is 73% greater in women than men [15]. Elderly people are a vulnerable group, and long questionnaires may be perceived as tiresome to fill in, not only by the elderly individuals themselves but also by health care personnel. A short screening instrument that could identify persons with self-reported insomnia would therefore be useful.

The Minimal Insomnia Symptom Scale (MISS) was developed from a more extensive sleep questionnaire and is a brief, three item screening instrument [1]. The basic psychometric properties of the MISS were evaluated in a randomly selected general population (n = 1075) aged between 20 and 64 years, and the prevalence of insomnia was found to be 22.5%. Although the findings support the measurement properties of the MISS as an insomnia screening instrument, the extent to which these data are applicable to an elderly population is still unknown. Our aim was to test the measurement properties of the MISS among people aged 65 + in Sweden, by replicating the original study [1] in an elderly sample.

## Methods

### Design and study sample

The study, which had a cross-sectional explorative design, was conducted within the *Swedish National Study on Ageing and Care - Blekinge *(SNAC-B) longitudinal study, which is part of the Swedish National Study on Ageing and Care [16]. The SNAC-B population consists of elderly persons living in Karlskrona municipality, which is situated in the south-east of Sweden and comprises both urban and rural areas. When the SNAC-B was launched in 2001, 1402 persons agreed to participate. The SNAC-B target sample, 59.4% of which were women, included ten age clusters representing the ageing population of Sweden. There was a randomized selection of the younger age clusters (60, 66, 72 and 78 year olds) as well as a total inclusion of the clusters of people aged 81, 84, 87, 90, 93 and 96 years at baseline [16]. In 2008, when the data collection for the present study took place, 978 persons were still alive and registered as participants, of whom 892 were available for participation.

The original ten age clusters used in SNAC-B were amalgamated into three clusters for this study; 65-74 years (including the 67 and 73 year olds), 75-89 years (including the 79, 85 and 88 year olds) and 90+ (including those aged 91 years or older). The distribution of individuals across the age clusters and in terms of gender is described in Table 1. Approximately 19.1% of the population of Karlskrona municipality is 65 years or older, which is a slightly higher age composition compared to Sweden as a whole [16].

**Table 1:** Distribution of the study sample.

Age-group	Male (%)	Female (%)	Total (%)
65-74	119 (51.1)	146 (46.3)	265 (48.4)
75-89	92 (39.5)	131 (41.6)	223 (40.7)
90+	22 (9.4)	38 (12.1)	60 (10.9)

**Total**	**233 (42.5)**	**315 (57.5)**	**548 (100)**

### Procedure

The study was approved by the regional Ethical Review Board in Lund (LU 605-00, LU 744-00, 178/2008). In December 2008, questionnaires were mailed to the 892 persons participating in the SNAC-B study together with a consent form and a pre-stamped response envelope. Six weeks later, a reminder was sent to those who had not yet returned the questionnaire. All participants had the possibility to withdraw from the study at any time without having to give a reason and were assured of confidentiality.

### Questionnaire

The survey consisted of the Uppsala Sleep Inventory 25 (USI-25) and three additional questions concerning age, sex and ability to fill out the form (independently, with help from a relative/friend or home care personnel).

The USI-25 is a 25 item self-report questionnaire about sleep difficulties and habits [1] previously used in studies of sleep associated with specific health conditions [17-19], among the elderly [20] and in longitudinal studies [21,22]. The USI-25 includes MISS, which consists of three items describing the major features of insomnia, i.e. difficulties initiating sleep, waking at night and not feeling refreshed by sleep[1]. Each item has five response alternatives; no problem (0), small problems (1), moderate problems (2), severe problems (3) and very severe problems (4). This yields a total score ranging between 0 and 12, where higher scores indicate more severe insomnia. A cut-off score of ≥6 on the MISS has previously been suggested for identifying insomniacs in the general adult population between 20 and 64 years old (sensitivity 0.82; specificity 0.86; positive predictive value 0.44; negative predictive value 0.95). The average item-total correlation was 0.55 and reliability (coefficient alpha) was 0.73. Validation against self-reported indices of insomnia criteria (confirmatory answers about suffering from sleep difficulties at least three times per week for a minimum duration of one month in addition to major daytime impairments due to poor sleep) supported the instrument's validity [1].

### Data analysis

Analyses were conducted using SPSS 17.0, (SPSS Inc Chicago, IL.) P-values < 0.05 were considered statistically significant.

First, assumptions concerning adding up item scores into a total score in accordance with the Likert tradition were tested, i.e. item mean scores, standard deviations and corrected item-total correlations should be fairly similar across items. Furthermore, there should be evident that items represent a common variable. This was considered supported if corrected item-total correlations were ≥0.4 [23]. Floor and ceiling effects (i.e., the proportion of people with minimum and maximum scores respectively) and score reliability (coefficient α) were then calculated. Floor and ceiling effects should not exceed 15% [24] and reliability should be between 0.7 and 0.9 [25].

We then examined the ability of the MISS scores to identify persons with self-reported insomnia and explored different cut-off scores. A criterion variable was constructed by amalgamating three USI-items regarding daytime sleepiness, feeling physically tired after sleep and experiencing sleep difficulties. This variable served as a proxy gold standard against which the MISS scores could be compared. Respondents who reported the presence of severe or very severe problems on these items were considered to suffer from insomnia. Although our criterion variable differs from that used in the original MISS publication [1], it agrees with some of the insomnia criteria described in the ICSD-2, such as daytime sleepiness, fatigue and concerns about sleep [3]. A receiver operating characteristic (ROC) curve was then drawn and the area under the ROC curve (AUROC) was calculated. The ROC-curve illustrates the discriminating abilities of a test at different cut-off values [26]. Based on the ROC-curve, sensitivity and specificity were calculated for various cut-off scores. Sensitivity is the ability to diagnose a condition correctly, while specificity measures the ability to accurately identify non-cases. These values provide an indication of the discriminatory abilities of the scale [27]. In addition, the positive predictive value (PPV) and negative predictive value (NPV) were calculated. These values depend not only on sensitivity and specificity but also on the prevalence of the condition in question [27,28]. PPV and NPV represent the probability of a certain outcome when the screening results (i.e. whether scores are above or below a certain cut-off) are known.

When sensitivity and specificity are considered equally important, Youden's index (sensitivity + specificity -1) can be used to choose an appropriate cut-off value. Since the maximum value of Youden's index is 1, indicating a perfect test, the cut-off score associated with the highest Youden index (J) is considered optimal [29]. Finally, the prevalence of insomnia (including 95% confidence intervals) according to the MISS was determined.

## Results

### Description of the study sample

**Figure 1:**
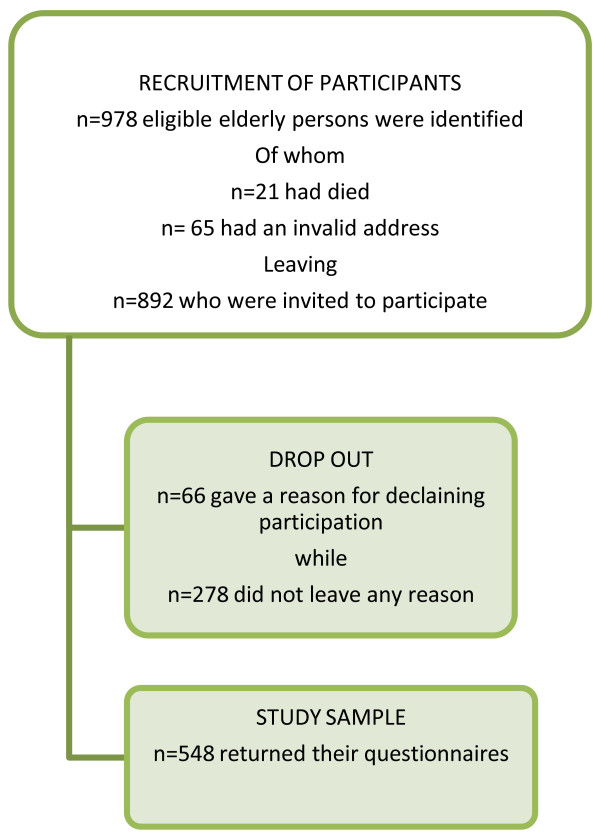
**Invited participants and drop-outs**.

Of the 892 questionnaires, 548 were returned together with a signed informed consent form, yielding a response rate of 61.4% (Figure 1). The mean age of the respondents was 77.8 (SD 8.63) years. The distribution of respondents across genders and the three age clusters are presented in Table 1. Common reasons for declining participation were advanced age, illness, vision disability, being unable to obtain help to fill in the questionnaire or feeling tired. The external drop out (Table 2) mainly represents persons of advanced age and those with cardiovascular disease or type-2 diabetes. Most of the participants (n = 479; 87.5%) completed the questionnaire on their own, while 66 (12%) obtained help from a relative or friend and three (0.5%) received assistance from health care personnel.

**Table 2:** Characteristics of non-participants vs. participants.

Variables	Non-participants (n = 344)	Participants (n = 548)	**p-value**^**1**^
Age mean (SD)	83.2 (9.8)	77.8 (8.6)	< 0.001
			
Sex			0.302
*Female %*	39.9	60.1	
*Male %*	36.2	63,8	
			
Angina Pectoris %	48.8	51.2	0.014
			
Arthritis %	43.0	57.0	0.183
Cancer %	43.0	57.0	0.328
Chronic heart failure %	62.2	37.8	0.001
			
Depression %	43.9	56.1	0.236
Diabetes type 2 %	53.8	46.2	0.012
Hypertension %	44.8	55.2	0.019
			
Osteoporosis %	55.2	44.8	0.084

^1^) Chi-square test and Mann-Whitney U-test. For the Chi-square test Yates' correction has been used.

Internal drop-out on variables was 0-1.2% accept for osteoporosis (10.5%) and arthritis (17.1%).

Responses to the USI-25 items revealed that close to a third (28.5%) of the sample considered themselves to suffer from sleep difficulties (9 persons failed to answer this question). Regular use of sleep medication was found to increase with age; 5.0% of those aged 65-74 years used sleep medication very often, while the corresponding figure among the oldest old was 24.1%. There was also a predominance of women among sleep medication users. The highest proportion of daytime impairments (i.e., daytime sleepiness, feeling physically tired after sleep and reporting sleep difficulties) was found among the 65-74 year olds (7.1%), whereas only 3.8% of the oldest old reported such impairments. Interestingly, of those who claimed to have no sleep difficulties, 317 reported insomnia symptoms on the MISS. There were more women than men that reported insomnia symptoms (Table 3).

**Table 3:** Sleep difficulties found among the elderly persons.

Sleep difficulty	Female	Male	65-74	75-89	90+
Difficulties initiating sleep %	18.6	9.1	13.8	15.5	13.8
Difficulties maintaining sleep %	22.3	15.6	19.5	18.7	21.4
Not being refreshed %	18.2	12.8	17.8	14.2	13.2
No insomnia symptoms %	5.4	8.3	5.8	8.9	1.9
One insomnia symptom %	16.8	12.0	13.4	16.4	13.2
Two insomnia symptoms %	10.4	6.9	9.1	8.9	7.5
Three insomnia symptoms %	6.4	3.7	6.2	3.9	5.7

Insomnia symptom = reports of severe/very severe problems with initiating sleep, maintaining sleep or not being refreshed by sleep.

### Measurement properties of the MISS

Fifty-one persons (9.3%) failed to fill in all the MISS items, thus complete MISS data were obtained from a total of 497 individuals. Mean MISS item scores ranged between 1.2-1.7 (SD 1.0-1.2) and corrected item-total correlations between 0.64-0.70. Inter-item correlation ranged between 0.55-0.62. Reliability was 0.81. Floor and ceiling effects were 6.6 and 0.6%, respectively.

**Figure 2:**
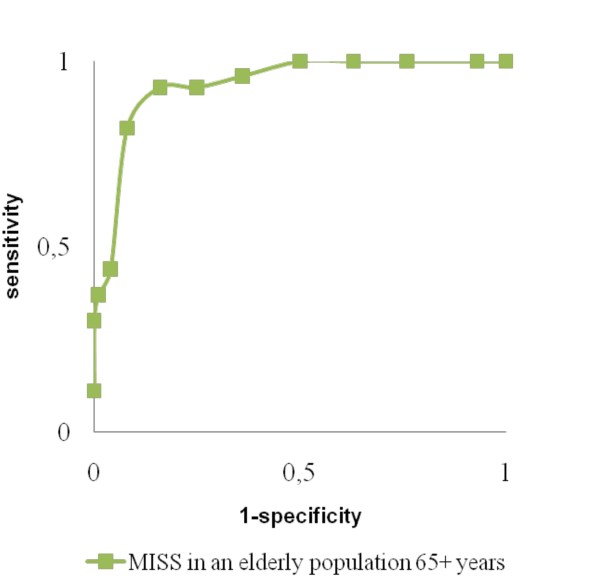
**Receiver operation characteristic curve**.

The criterion validity of the MISS was tested against the amalgamated criterion variable which corresponds with some of the diagnostic criteria in the ICSD-2 [3]. The MISS ROC-curve is presented in Figure 2. The area under the curve was 0.935 (95% CI: 0.90-0.97; P < 0.001), indicating good correspondence between the criterion variable and the MISS. According to the Youden index, a cut-off MISS score of ≥7 was found the most optimal (Table 4).

**Table 4:** MISS cut-off values, sensitivity, specificity, positive predictive value (PPV), negative predictive value (NPV) and Youden's index.

Cut-off score	Sensitivity	Specificity	1-specificity	PPV	NPV	Youden's index
≥5	0.963	0.643	0.357	0.137	0.997	0.606
≥6	0.926	0.752	0.248	0.181	0.994	0.678
≥7	0.926	0.838	0.162	0.256	0.995	0.764
≥8	0.815	0.920	0.080	0.377	0.988	0.730

### Prevalence of insomnia

The mean MISS score for the total sample was 4.11 (95% CI, 3.86-4.35). The prevalence of self-reported insomnia in this sample of elderly persons based on various MISS cut-off scores can be seen in Table 5. Using a cut-off of ≥7, the overall prevalence of insomnia was 21.7% (95% CI, 18-25%). Insomnia tended to be more common among persons aged 90 years or over (24.1%) and affected more women (26.8%) than men (15.2%). Among those identified as insomniacs, 12.3% answered "no" to the question of perceived sleep difficulties.

**Table 5:** Prevalence of insomnia according to different MISS cut-off scores.

Cut-off	*Mean score* *(95%CI)*	*All* *(n = 497)*	Male (n = 217)	Female (n = 280)	65+ (n = 242)	75+ (n = 201)	90+ (n = 54)
≥5	6.91 (6.67-7.15)	202 (40.6%)	69 (31.8%)	133 (47.5%)	99 (40.9%)	82 (40.8%)	21 (38.9%)
≥6	7.57 (7.33-7.81)	150 (30.2%)	51 (23.5%)	99 (35.4%)	71 (29.3%)	62 (30.8%)	17 (31.5%)
≥7	8.19 (7.94-8.44)	108 (21.7%)	33 (15.2%)	75 (26.8%)	52 (21.5%)	43 (21.4%)	13 (24.1%)
≥8	8.97 (8.68-9.26)	65 (13.0%)	21 (9.6%)	44 (15.7%)	32 (13.2%)	23 (11.4%)	10 (18.5%)

## Discussion

This study tested the measurement properties of a brief insomnia screening instrument, the MISS, among elderly persons. Previous testing of the MISS in the general population under 65 years of age, found it to be sound and capable of identifying insomnia. This study provides further support for the usefulness of the MISS and extends its application to include elderly persons. However, our findings differ somewhat from the previous study in terms of suggested cut-off score and diagnostic ability. Based on available MISS data we also consider possible developments of the MISS in order to enhance its usefulness, by specifying the response alternatives and add a fourth item comprising daytime impairments.

The screening capacity of the MISS in this study (AUROC, 0.94; 95%CI, 0.9-0.97) was similar to that reported in a younger adult population ( AUROC, 0.92; 95%CI, 0.89-0.94) [1]. But a cut-off value ≥7 was found more suitable than that of ≥6 previously identified in younger adults [1]. This divergence may be due to the variation in size and age of the study samples, as well as the use of different gold standards. It is possible that healthy, elderly persons perceive disturbed sleep and daytime impairments as related to the advanced age. The proxy-gold standard used in this study prerequisite recognition of daytime impairments. There is thus a risk that the prevalence of insomnia is under-recognized. This would also have bearing on the reported positive predicted value. Similarly to the previous study [1] MISS scores were not directly tested against diagnostic insomnia criteria but a proxy gold standard was used instead. However, the proxy gold standard was not the same as in the original study. While this may explain the observed differences in result, future studies should consider testing the MISS directly against diagnostic insomnia criteria in order to provide support for the legitimacy of creating a total MISS score that is reliable, unidimensional and valid in people aged 65 years or older.

A cut-off value of ≥7 revealed a 21.7% prevalence of insomnia in the present study. In previous testing of the MISS in the general population using a cut-off value of ≥6, the prevalence of insomnia was found to be 22.5% [1]. This shows a similarity between the studies in estimating the prevalence of insomnia symptoms. Other studies have found that insomnia symptoms occur in 33-50% of the adult population, while the prevalence of insomnia disorder has been estimated to about 10-30% [14,30-32]. In relation to these figures, the MISS appears to blunt for diagnosing insomnia.

Therefore, if the goal is to diagnose insomnia, additional investigations based on clinical diagnostic criteria are needed, as pointed out by the constructors of the MISS [1]. The MISS should thus be regarded as an initial screening instrument. Although its brevity is advantageous in the inclusion of only three items also limits the usefulness of the MISS. It appears reasonable to suggest that the MISS would benefit from adding a fourth item concerning daytime impairments which is among the diagnostic criteria for insomnia [3]. Similarly, it could also be considered to incorporate some references to the duration and frequency of the sleep difficulties specified by the MISS items, possibly by integrating this with the response options. Compared to other available short insomnia screening questionnaires, which contain 6-10 items [33-35], such an expansion of the MISS would still render the briefest insomnia screening questionnaire.

Healthy elderly persons adjust their expectations of sleep, accepting the changes as a normal part of aging. It has been suggested that it is the individual's perspective of growing old that is significant for the experience of good or poor sleep. The presence of a medical disease or chronic illness has stronger correlations with poor sleep than chronological age per se [36,37]. Women also appear to accept sleep loss as a natural part of life. Sociologists consider that women indeed recognize sleep as essential for good health, but the demands of their social roles determines to which extent they can access adequate sleep. Over time the social roles in life of the women, conceal the sleep disruption until poor sleep patterns becomes the norm [38]. This could imply that women and elderly persons are inattentive to insomnia symptoms, contributing to the under-diagnosis of the disorder. Our findings reveal that insomnia is more prevalent in women and in the oldest old. However, the MISS is a self-report questionnaire, reflecting subjective experiences rather than the prevalence of insomnia disorder, which is problematic when comparing groups. Objective measures could yield a different result [39]. Furthermore, previous research has found that self-reports of polysomnographically measured sleep are less accurate and valid in older persons compared to younger [36].

## Limitations

There are limitations in the design of the study that need to be considered. A concern arose when using the USI-25 questionnaire, particularly when trying to validate the MISS, as the former contains no measure of the duration or frequency of sleep difficulties, nor daytime impairments. This makes it difficult to establish a clinical diagnosis of insomnia, as such a diagnosis is based on the duration of the sleep difficulty, impaired daytime functioning and the three major symptoms [3]. However, it was considered important not to increase respondent burden by using multiple questionnaires, due to the vulnerability of the study sample and the risk of further compromising the response rate.

Another limitation was the cross-sectional design. Repeated measures could have contributed to more robust reliability and validity [40]. Furthermore, the study design did not allow for any testing of sensitivity to change in order to verify or reject previous findings [1], which have provided some support for responsiveness of the MISS.

As the respondents participated in the SNAC-B study, we had access to data about the prevalence of different diseases in the sample, which was used when analysing the external drop out. The external drop-out analysis revealed that persons with cardiovascular disease and diabetes, who could be expected to suffer from insomnia [41,42], chose to decline participation, which may have influenced the findings. Furthermore, the MISS has not been tested as an independent instrument but was extracted from the USI-25 questionnaire, which may have implications for the response rate in the present study. Finally, future studies on the MISS should consider using more advanced psychometric methods, such as Rasch analysis or item response theory.

## Conclusion

This is the first study of the measurement properties of the MISS in an elderly population, and our observations provide support for its reliability and validity in elderly persons in Sweden. As such, the results provides further support that the MISS is useful for identifying elderly persons with insomnia-like sleep problems who would be candidates for more detailed assessments. However, further studies are needed to assess its usefulness in identifying clinically defined insomnia.

## Competing interests

The authors declare that they have no competing interests.

## Authors' contributions

AH was responsible for data collection, data analysis and drafting of the manuscript. PH contributed to the design and conception of the study, statistical expertise, critical revision and important intellectual content. CF helped with the statistics, the design and conception of the study and also performed critical revisions and supervision. AW was involved in the drafting of the manuscript, critical revisions, important intellectual content and supervision. All of the authors have read and approved the final manuscript.

## Pre-publication history

The pre-publication history for this paper can be accessed here:

http://www.biomedcentral.com/1471-2318/10/84/prepub
